# Extrarenal expression of *α-klotho,* the kidney related longevity gene, in *Heterocephalus glaber,* the long living Naked Mole Rat

**DOI:** 10.1038/s41598-021-94972-1

**Published:** 2021-07-28

**Authors:** M. Morevati, M. L. Mace, S. Egstrand, A. Nordholm, C. Doganli, J. Strand, J. L. Rukov, S. B. Torsetnes, V. Gorbunova, K. Olgaard, E. Lewin

**Affiliations:** 1 Nephrological Department P 2131, Rigshospitalet, University of Copenhagen, 9 Blegdamsvej, 2100 Copenhagen, Denmark; 2 Nephrological Department B, Herlev Hospital, University of Copenhagen, Copenhagen, Denmark; 3 Department of Cellular and Molecular Medicine, University of Copenhagen, Copenhagen, Denmark; 4 Randers Regnskov, Randers, Denmark; 5 Department of Neurology, Akershus University Hospital, Oslo, Norway; 6 Department of Biology, University of Rochester, Rochester, NY USA

**Keywords:** Cell biology, Molecular biology, Nephrology

## Abstract

The Naked Mole Rat (NMR), *Heterocephalus glaber*, provides an interesting model for studying biomarkers of longevity due to its long lifespan of more than 30 years, almost ten times longer than that of mice and rats. *α-Klotho* (*klotho*) is an aging-suppressor gene, and overexpression of *klotho* is associated with extended lifespan in mice. *Klotho* is predominantly expressed in the kidney. The expression profile of *klotho* in the NMR has not previously been reported. The present investigation studied the expression of *klotho* in the kidney of NMR with that of *Rattus Norvegicus* (RN) and demonstrated that klotho was expressed in the kidney of NMR at the same level as found in RN. Besides, a significant expression of *Kl* mRNA was found in the liver of NMR, in contrast to RN, where no hepatic expression was detected. The Klotho expression was further confirmed at the protein level. Thus, the results of the present comparative study indicate a differential tissue expression of *klotho* between different species. Besides its important function in the kidney, Klotho might also be of significance in the liver of NMR. It is suggested that the hepatic extrarenal expression of *klotho* may function as a further longevity-related factor in supplement to the Klotho in the kidney.

## Introduction

The Naked Mole Rat (NMR) is an eusocial, subterranean rodent that lives more than 30 years, which is almost ten times longer than mice and rats in captivity (app. 3–4 years) or of any other small rodent^1,2^. In the past few years, the NMR has been used as a model for longevity studies due to its lifespan, exceptional features and extraordinary biology, that has been adapted as a consequence of the challenges posed by a subterranean niche^3,4^. The NMR is a mouse-sized (approximately 35 g in weight) rodent, which does not show any increase in the age-specific hazard of mortality, in defiance of Gompertzian laws^5^. This species shows no decrease in physiological capacity, cardio-vascular function, gastrointestinal function, glucose tolerance, and reproductive capacity until the third decades of life^6,7^. NMR is further characterized by resistance to sarcopenia and potential low susceptibility to cancer^8–11^. The longevity of NMR, together with their low growth rate, might be a consequence of a very high energy consumption in these rats as they are exposed to high levels of oxidative stress early in life^12^.

α-Klotho (in the following called Klotho) is an evolutionarily, highly conserved kidney hormone, that is related to longevity^13,14^. It is distinctly expressed in the kidney and found in small amounts in the parathyroid glands and the plexus choroideus in the brain^13,15^. The Klotho gene (*Kl)* is located on chromosome 13 in humans and 12 in rats and contains five exons and four introns. In humans and mice, along with the full-length transcript of *membrane-bound α-klotho (mKl)*, an alternatively spliced Klotho has also been identified as a nonsense-mediated mRNA decay target^16,17^, known as *sKl.*

Klotho has a critical role in longevity and healthy aging^18^. This is partially effectuated by its circulating forms that are excreted into serum, urine, and the cerebrospinal fluid, and which might have a direct effect on tissues or cells that do not express *klotho* (e.g. vascular endothelial and smooth muscle cells)^16,19–23^.

Knockout of *klotho* in mice results in a shortened lifespan, and *klotho* overexpression in transgenic mice extends the lifespan by 30%^14,24^. The *klotho* deficient mice develop accelerated aging features besides shortened lifespan, organ and tissue atrophy, osteoporosis, sarcopenia, frailty, and a severe vascular phenotype of calcification, arteriosclerosis, and impaired endothelial function^13,25^. Klotho is connected to functions that protect the vasculature against osteochondrocytic conversion, including inhibition of high phosphate (P) induced vascular calcification through inhibition of the sodium dependent phosphate transporters, Pit-1 and Pit-2, or by Wnt antagonism^26–28^. In humans circulating levels of Klotho decrease with age^13,29–31^. Reduction in Klotho levels has also been proposed in patients with several aging-related diseases such as cancer, Alzheimer’s disease, hypertension, and uremia^32–36^. Measuring of plasma Klotho protein levels is however, difficult due to lack of good antibodies and reliable commercial assays^37^.

Klotho is an important renal hormone involved in the calcium (Ca) and P homeostasis as an obligatory co-receptor for the action of fibroblast growth factor 23 (FGF23) in the kidney via the fibroblast growth factor receptor (FGFR)^13,29,38^. FGF23 decreases the type II sodium-dependent P co-transporters (NaPi2a and NaPi2c) in proximal tubules, and is thereby inhibiting P reabsorption^39^. In NMR plasma P levels has been found to be considerably lower as compared to that of Rattus Norvegicus (RN) and other small rodents. Furthermore, an inverse correlation has been demonstrated between plasma P and longevity in mammals indicating a relationship between longevity and regulators of mineral homeostasis^40^.

FGF23 and thus Klotho reduce the synthesis of 1,25 (OH)_2_ vitamin D by inhibition of the 25-OH vitamin D 1α-hydroxylase (Cyp27b1) and by stimulation of 1,25 (OH)_2_ vitamin D 24α-hydroxylase (Cyp24a1) via the KL/FGFR-ERK pathway^41^. Recently, the crystal structure of the KL-FGFR1c-FGF23 complex has been resolved, providing a structural basis for understanding the unique feature of FGF23 and its requirement for Klotho to exert their hormonal actions^42^. Thus, Klotho enhances the renal activity of FGF23, and P depletion has been shown to rescue most of the aging phenotypes of *klotho* knockout mice^43,44^. The physiological and biochemical factors responsible for these effects are not well characterized, and it is not known whether the long-living NMR represents a specific expression of the anti-aging and longevity hormone, Klotho^45^.

In the present investigation, we therefore examined the level of expression of Klotho in the kidney of NMR and further investigated whether a potential kidney expression of Klotho was increased, as compared to that of the common rat, RN. Our comparative study further examined whether Klotho was expressed in other tissues (liver, lung and skin) of the long-living NMR versus RN. The hypothesis was that the longevity of NMR was associated with increased expression levels of Klotho.

## Methods

### Animals and study design

The Naked Mole Rat belongs to a very selective group of protected mammals, which can’t be bought commercially for experimental research*.* Kidney, liver, skin, and lung samples from five redundant NMRs were kindly obtained from a Danish Zoo-housed population (Randers Regnskov, Randers, Denmark). The genders and ages of these animals were unknown. Another five NMR sets of organs were kindly provided as specimens from a research colony at the Gorbunova and Seluanov laboratory (Aging Research Center, University of Rochester, USA). This was a mix of both genders and different ages. In comparison, corresponding tissues from adult male Wistar rats (Taconic, Ejby, Denmark) with a weight of 200 g were used.

The experimental protocols were approved by the Danish Animal Experiments Inspectorate, Ministry of Environment and Food of Denmark, the Danish Veterinary and Food Administration (reference no: 2017-15-0201-01214) and performed in accordance with the National Institute for Health’s Guidelines for the Care and Use of Laboratory Animals. The study was carried out in compliance with the ARRIVE guidelines.

### Quantitative PCR (qPCR)

The current genome of NMR is not completely annotated. Therefore, “in silico” sequence alignment was used to identify the right sequence for *klotho*. The genomic sequence of *klotho* was obtained from the Ensembl genome browser for Human and Rat and from NCBI genome browser for NMR (Table 1). The coding region of m*Kl* for all three species was manually aligned to identify areas of highest homology. Feasible exons within the regions of high homology and where the alternative isoform for *klotho* exist in human and mice kidney^17^ as well as in the brain of NMR^45^ were then selected as target areas for design of qPCR and PCR primers for NMR and RN (Table 2).

**Table 1:** Genome assemblies used for klotho.

Species	Genome assembly
Homo Sapiens	December 2013 (GRCh38/hg38)
Rattus Norvegicus (RN)	July 2014(RGSC 6.0/rn6)
Heterocephalus Glaber (NMR)	February 2012 (Broad Institute HetGla_Female_1.0/hetgla2)

**Table 2:** Primer sequences for short PCR and qPCR.

Primer sequences for qPCR		
Name	Gene symbol	Sequence 5′–> 3′
α-klotho	*RN-Kl*	F: CGTGAATGAGGCTCTGAAAGC R: GAGCGGTCACTAAGCGAATACG
	*NMR-Kl_1*	F: GTTGACAACTACATTCAAGTAGAC R: TGCTTCTTGGCTGCAACTCC
	*NMR-Kl_2*	F: CTTGCAGGCTGATTGGATAGA R: GCCAGCCAATGTCAAATTCC
Reference genes		
Ribosomal protein l13a	*RN-rpl13a*	F: CCCTCCACCCTATGACAAGA R: CCTTTTCCTTCCGTTTCTCC
	*NMR-rpl13a*	F: CCCGCCACCCTATGACAAGA R: CCTTCTCCTTCCTTTTCTCC
Eukaryotic translation initiation factor 4a2	*NMR- and RN-elf4a2*	F: GACAGCCACATTTGCTATTTCC R: GGATCTGTTGAGCCAGTTCTC

Tissues were manually grounded and placed in liquid nitrogen. Total RNA was extracted from the tissue-powder using the EZNA RNA kit (Omega Bio-Tek, GA, USA). The first-strand cDNA was synthesized from 1.5 μg of RNA with Superscript III cDNA kit (Invitrogen, MA, USA). Jumpstart (Sigma-Aldrich, MO, USA) and LightCycler 480II (Roche, Basel, Switzerland) were used for qPCR. The cDNA levels were normalized to the mean of reference genes (Table 2), which were selected according to the stability using geNorm software^46^.

### Polymerase chain reaction (PCR)

PCR amplification of *mKl* cDNA from a pool of five kidney samples and five liver samples was performed by Taq DNA polymerase (Thermo Scientific, EP0281) following the manufacturer’s protocol. Amplification of a long fragment of *mKl* cDNA was performed by one pair of primer (Kl-FOR_1/Kl-R2) (Table 3) using the following conditions: initial denaturation at 95 °C for 1 min, followed by 30 cycles, each consisting of denaturation at 95 °C for 30 s, annealing at 56 °C for 42 s and extension at 72 °C for 42 s with a final extension at 72 °C for 10 min. The PCR product was loaded on a 1.5% agarose gel, and the band with the right size (2357 bp) corresponding to the *mKl* transcript was cut out. The QIAquick Gel Extraction Kit (# 28704) was used according to the manufacturer’s protocol for cleaning the band from the agarose gel.

**Table 3:** Long-range PCR and sequencing primers.

PCR and sequencing primer			
Naked Mole Rat α-klotho			
Primers	Sequencing range	Start site	Sequence 5′–> 3′
*Kl_FOR_1*	701–1400	654	ATCACGATCGACAACCCCTA
*Kl-FOR_2*	1401–2100	1354	CCAACGTTTACCTGTGGGAC
*Kl-FOR_3*	2101–2763	2048	GGATTGGCTGAACCAAAGAA
*Kl-REV_1*	700–1	753	CCACCAGGTACCCGAGCC
*Kl-REV_2*	1400–701	1439	GCAGTAGGATTTCCGCTTCTT
*Kl-REV_3*	2100–1401	2154	TGGTGTAATGGCTTAGGGCT
*Kl-R2*	2063–2763	2763	CTATTTGTAACTTCTTCTGCCTTTC

### Sanger sequencing

The cDNA from 5 NMRs was pooled together for kidney and liver, respectively, and amplified by PCR, as described above. The quality of the PCR product was confirmed on 2% agarose gel. The amplicons were then sequenced in both directions using BigDye Terminator version 1.1 Cycle sequencing kit (Life Technologies) and sequenced on ABI 3130XL genetic analyzer by BigDye 3.1. Sequence traces were aligned to the NMR (XM_021251513.1) gene reference sequence. Nucleotide BLAST Program (http://blast.ncbi.nlm.nih.gov/) was used to align sequences to check possible identical sequences between proximal and distal breakpoints. Primers used for Sanger sequencing are listed in Table 3.

### In silico analysis

In order to analyze possible cleavage recognition sites in Klotho of NMR alignment of NMR Klotho amino acid sequence (Query_5384: ENSHGLP00000007318 Ensembl Translation) with Rattus Norvegicus (BAA34740.1: NBCI) was performed.

### Targeted liquid chromatography–mass spectrometry analysis

No specific antibodies exist for detecting Klotho in NMR. We therefore examined the protein expression of Klotho in NMR, in four kidneys and four livers by proteomics analysis using Parallel Reaction Monitoring (PRM) at Biogenety, Aalborg, Denmark. PRM is an ion monitoring technique based on high-resolution and high-precision mass spectrometry, which can detect peptides with an attomole-level in complex samples^47^. Endogenous peptides that are quantifiable surrogates of Klotho were selected from the first domain of Klotho protein, KL1 (Supplementary Fig. 3). Since the protein sequence of NMR Klotho is not annotated, the unique peptides were designed to detect predicted NMR Klotho protein (Ensemble; ENSHGLT00000007403.1). Kidney and liver tissue from NMR were prepared with an iST kit from (Preomics, Germany). The recommended protocol for these tissues was used for lysis, digest and sample cleanup. The iST prepared samples were then analyzed using a Q-Exactive HF-X mass spectrometer (Thermo Fisher Scientific), which was operated in Parallel Reaction Monitoring (PRM) mode for 70 min. For sample injection, two μL of the sample (500 ng total protein) was picked up and loaded into the trap column. MS1 resolution of 70.000, AGC target set to 3*e6, maximum injection time set to 20 ms, scan range 350 to 1300 m/z for a full MS scan. MS2 scans used 30.000 resolution, AGC target of 2*e6, maximum injection time 100 ms, and isolation window of 1.2 m/z at normalized collision energy of 27, using several target peptides, where LDGVDVIGYTAR and LQDTYGGWANR worked best. Synthetic peptides for each target peptide were obtained from JPT technologies and injected in the initial run to confirm the elution patterns and determine the limits of detection. Peptides with a charge from + 2 to + 5 were selected for fragmentation.

### Statistical analyses

Normal distributed data are presented as mean ± standard deviation (SD). Statistical significance was tested using unpaired two-tailed t-test calculated in GraphPad Prism 8.0 to compare means between groups. Significance level was set at *p* ≤ 0.05.

## Results

### Confirmation of the specificity of NMR

**Figure 1:**
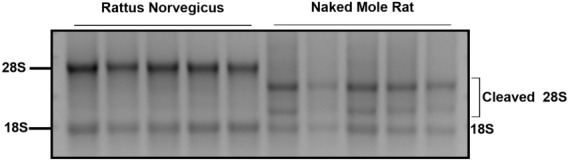
Characterization of the Naked Mole Rat. Detection of the fragmented pattern of 28S ribosomal RNA (rRNA) is characteristic for the NMR. The agarose gel electrophoresis of total RNA from RN and NMR liver is shown and the 28S and 18S of rRNA are indicated. NMR rRNA shows a fragmented pattern, where the 28S rRNA appears cleaved. NMR: Naked Mole Rat; RN: Rattus Norvegicus.

The NMR is characterized by a cleaved 28S ribosomal RNA (rRNA) fragment^48^. The specificity of the samples used in the present study was examined by electrophoresis of total RNA following by staining with ethidium bromide. The samples belonging to the NMR expressed a cleaved 28S characteristic for this species, in contrast to uncleaved 28S of rRNA in RN, confirming that the tissues used belonged to NMR (Fig. 1).

### *Klotho* expression in the kidney of NMR versus RN

**Figure 2:**
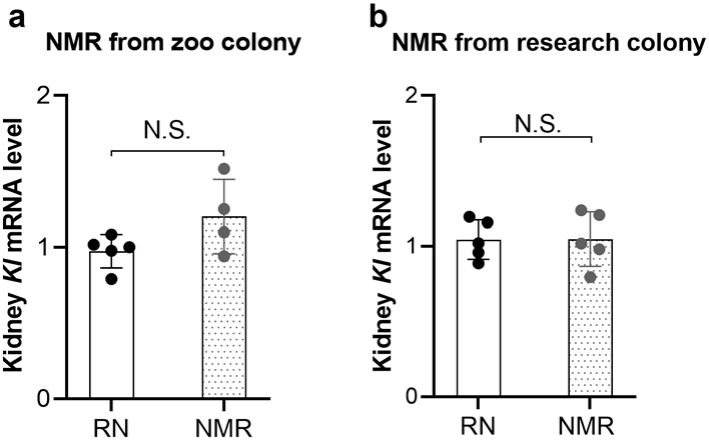
Expression of *klotho* in kidney from the Naked Mole Rat versus Rattus Norvegicus. Relative expression of *Kl* in kidney tissue from the NMR versus RN was measured by qPCR. The *Kl* expression was quantified and normalized to reference genes in the two experimental groups of NMRs (obtained from a zoo- and a research-colony) (**a**, **b**). Similar levels of *Kl* mRNA were found in the kidney of RN and NMR. Mean ± SD. *p < 0.05. *n* = 4–6 in each group. NMR: Naked Mole Rat; RN: Rattus Norvegicus; *Kl*: *klotho*.

The expression profile of *klotho* in NMR tissues was examined by designing specific primers for amplification of *mKl* and reference genes, as the NMR rats do not share the same sequence as RN (Table 2). This was initiated by examining the kidney tissue, as the kidney previously has been shown to be the primary source of *klotho* expression in mammals. qPCR analyses were performed on kidney tissues of NMR from both the zoo (Randers Regnskov, Denmark) and the research colony population (Gorbunova and Seluanov laboratory). The expression of *klotho* in the kidney of NMR was similar to that of RN (Fig. 2).

### *Klotho* expression in the liver of NMR

**Figure 3:**
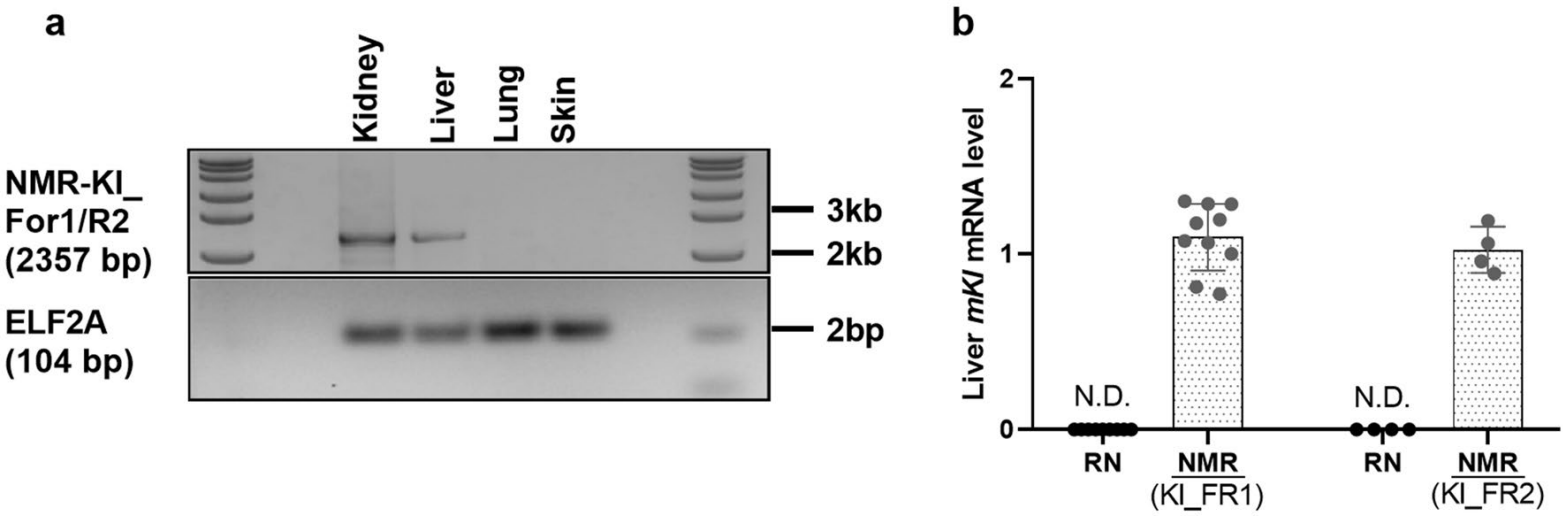
Expression of *klotho* in the liver of the Naked Mole Rat. The potential extrarenal expression of *Kl* in NMR was examined in the liver, lung, and skin by PCR on a long fragment of the coding region of NMR *Kl* (2357 bp). Each lane consists of pooled tissues from five rats (**a**). The relative mRNA expression of *Kl* in the liver was further measured by qPCR using two different primers (Kl_FR1 and Kl_FR2) for NMR and compared to that of RN. The *Kl* expression was quantified and normalized to reference genes. In NMR, a high expression of *Kl* mRNA was detected in the liver (*p* < 0.0001), while no *Kl* was found in the liver of RN (**b**). N.D.: not detectable. *n* = 5–12 in each group. NMR: Naked Mole Rat; RN: Rattus Norvegicus; *Kl*: *klotho*.

The expression of *klotho* was besides in the kidney examined in other organs, the lungs, skin, and liver of NMR by targeting a 2357 bp (643–3000 bp) region of the coding sequence by PCR. No expression *of klotho* was detected in the lung and skin of NMR, while a clear expression of *klotho* was observed in the liver of NMR (Fig. 3a). The hepatic expression of *klotho* was further examined by qPCR, and a high level of *klotho mRNA* was found in the liver of both the zoo-housed and research colony NMR in contrast to RN, where no expression of *klotho* was detected in the liver (Fig. 3b). Two different sets of primers, specific for NMR *mKl,* provided similar results and confirmed the presence of a significant expression of *Kl* mRNA in the liver of NMR.

### Identification of *mKl* in the liver of NMR by Sanger sequencing

The long fragment of the amplified band for *mKl* (2357 bp) in the NMR kidney and liver was amplified (Supplementary Fig. 1) and sequenced to verify the *mKl* transcript in the liver. As Sanger sequencing can only sequence less than 1000 bp, six other short primers were designed by GenScript with a dispense of 400 bp between sequencing primers (Table 3). The sequenced fragments of the liver were aligned to the sequenced fragments of *mKl* from the kidney (Supplementary Fig. 2), which previously has been confirmed to represent *mKl* by aligning to the published sequence from NCBI. The sequenced *klotho* in the liver was identical to that found in the kidney. Thus, these results clearly indicate that the liver of NMR expresses *mKl*.

### *Klotho* and the alternative splicing site in the liver of NMR

**Figure 4:**
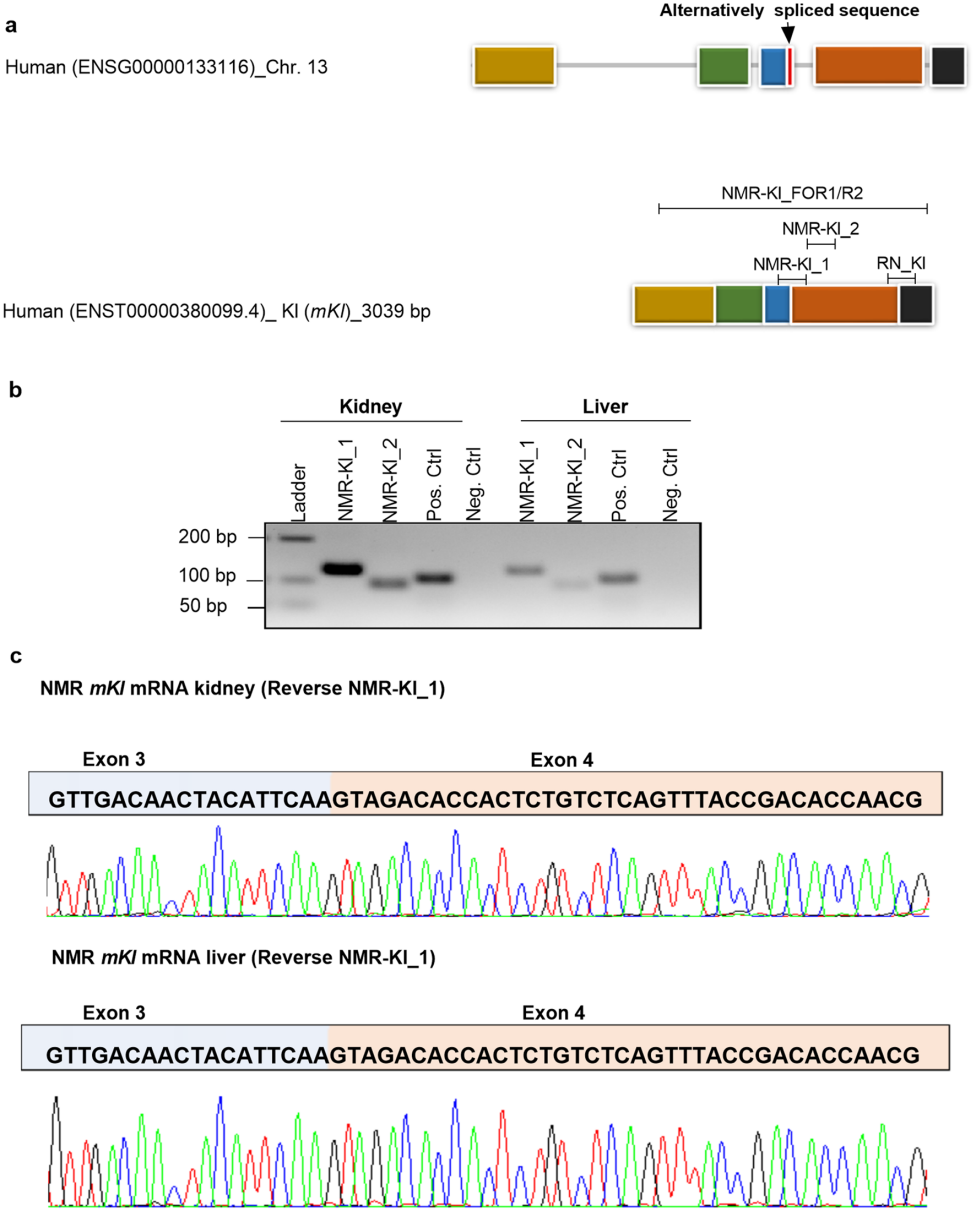
Identification of *Kl*-mRNA by PCR and Sanger sequencing in the Naked Mole Rat. (**a**) Architecture of the *Kl* in humans and locations of customized primers for the Naked Mole Rat (NMR) and Rattus Norvegicus (RN). The primers were designed in order to measure the expression of *mKl* in NMR (NMR-Kl_1 and 2) and RN (RN_Kl), The designed primers, specific for RN and NMR *klotho* are visualized in relation to the simplified illustration of well characterized human mRNA sequence. Line-box diagram showing the exon–intron arrangement for Human *mKl*. Boxes represent exons, while the black line represents introns; exon 1: yellow, exon 2: green, exon 3: blue, exon 4: orange; exon 5: black. Two transcripts of *Kl* have been found in humans. The red box represents part of the intron between exon three and four that is alternatively spliced in humans, yielding mRNA transcript known as s*Kl***.** The black arrows denote the positions of the alternative spliced sequence. The location of the long-range PCR primer (*Kl_FOR1/ Kl_R2*) used for identification of NMR tissue *Kl* (shown in Fig. 3) is depictured**.** (**b**) Identification of *Kl* mRNA by PCR and Sanger Sequencing in the NMR. Agarose gel electrophoresis displays PCR amplification of *Kl* mRNA in the kidney and liver of NMR measured by two primer sets with annealing to different positions on *mKl*. PCR using *NMR-Kl_1* primer set spanning on part of exon 3 (corresponding to exon 3 and 4 in human, where alternative splicing has been found before) yields one PCR product, which indicates no alternative splicing in NMR liver and kidney. (**c**) Sanger Sequencing shows that the *Kl* sequence in the liver is identical to that of the kidney in NMR. NMR: Naked Mole Rat; RN: Rattus Norvegicus; *Kl*: *klotho*.

Previously, alternatively spliced *klotho* mRNA has been found in humans and mice with a stop codon located between exons 3 and 4^17^, which hereby is a target for nonsense-mediated mRNA decay. In order to ensure that the *klotho* mRNA detected in the liver of NMR was not the alternatively spliced *klotho*, we amplified and sequenced the region spanning on the potentially alternatively spliced site of the NMR liver *klotho* and compared it to the NMR *mKl* from the kidney (Fig. 4a)^17^.

The predicted region around the alternatively spliced sequence for human *klotho* was amplified by PCR in the liver and kidney of NMR, using two sets of *mKl* primers (*NMR-Kl_1* and *2*) with annealing to two different positions on the gene, and confirmed on agarose gel run together with controls (Fig. 4b). Both primers for *klotho* yielded only one PCR product in the kidney and liver of NMR. The *NMR-Kl_1* primer set showed the best efficiency, and its amplified PCR product was further used for sequencing of *klotho* from the kidney and liver of NMR. The sequenced regions in both tissues were identical, and no sign of double sequence traces was observed in this region, indicating that the alternatively spliced region known from human *klotho* was not present neither in the liver or kidney of NMR (Fig. 4c).

### Detection of Klotho protein in the liver of NMR by proteomics

**Figure 5:**
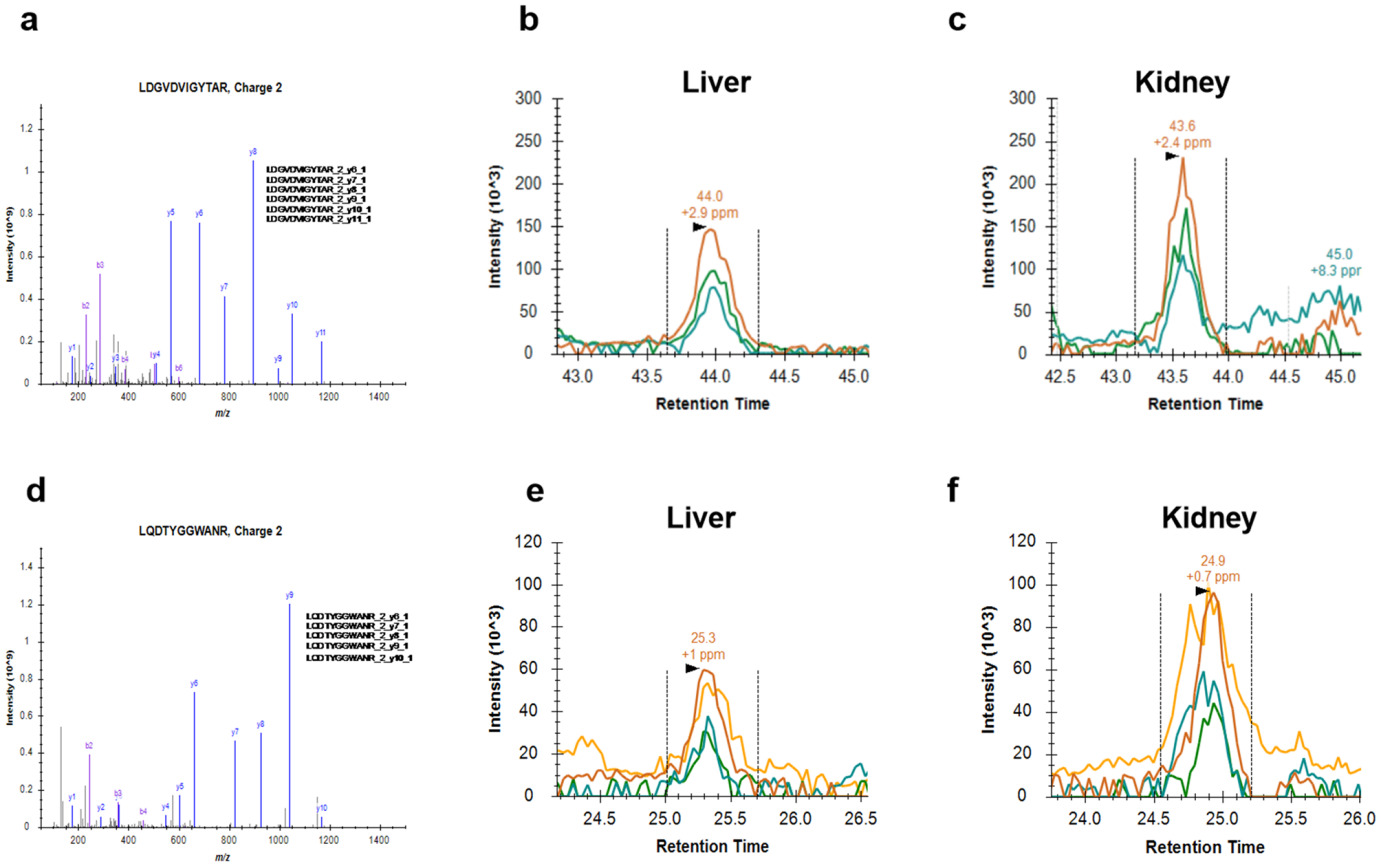
Detection of klotho protein in the liver of the Naked Mole Rat. Detection of klotho in the liver of the NMR with the kidney used as a positive control for klotho protein in Parallel Reaction Monitoring. (**a**) Mass spectrum with detected fragments obtained by analysis of a synthetic peptide (LDGVDVIGTAR), which is uniquely found in klotho. (**b**,**c**) Representative elution profile of the klotho unique peptide (LDGVDVIGTAR) in liver and kidney. (**d**) Mass spectrum with detected fragments obtained by analysis of a synthetic peptide (LQDTYGGWANR) and its fragments. (**e–f**) Representative elution profile of (LQDTYGGWANR), which is uniquely found in klotho in liver and kidney of NMR. n = 4 in each group. The detected mass deviation was less than 3 ppM. NMR: Naked Mole Rat.

The protein expression of Klotho in NMR was examined by proteomics analysis by Parallel Reaction Monitoring (PRM) in four NMR livers and kidneys (positive control). Since the protein sequence of NMR Klotho was not annotated, unique peptides were designed to detect predicted NMR Klotho protein. These peptides were aligned to human and rat Klotho protein and found identical. The peptides for Klotho detected by PRM are shown in Fig. 5a,d. A representative elution profile for the peptide “LDGVDVIGYTAR” in the liver and kidney is shown in Fig. 5b,c. The fragmentation ions for the peptide in the liver appeared in co-elution and with a shape (Fig. 5c) similar to the positive control, the kidney (Fig. 5b). The fragment relative intensities and retention times were also similar in the liver (44.0 min (+ 2.9 ppm mass error) and kidney (43.6 min (+ 2.4 ppm mass error) and close to the predicted retention time of synthetic peptides, which indicate detection of the same peptide in both tissues. The second peptide “LQDTYGGWANR” was also detected in both liver and kidney of NMR. The elution profile’s fragmentation ions were further observed in both tissues and with similar relative intensity and retention times of the fragments (liver 25.3 min (+ 1 ppm mass error) and kidney 24.9 min (+ 0.7 ppm mass error)), which again is similar to the predicted retention time of synthetic peptides (Fig. 5e,f). Thus, these results further indicate the existence of Klotho at the protein level in the liver of NMR.

### In silico analysis of the cleavage sites in Klotho of the Naked Mole Rats

Metalloproteases, mainly ADAM 10 and ADAM 17, have previously been shown to be responsible for shedding of Klotho^20^. Cleavages that produce the 130 kDa and 70 kDa soluble forms of Klotho were termed α and β-cut, respectively. Two potential recognition sites for sheddase on Klotho, at β and α-cut sites, have been identified. PPLPENQPLE recognition site sequence (between KL1 and KL2 domains) at the β-cut site is a conserved sequence in mice, rats, and humans. At the α-cut site (close to the transmembrane region), PGPETLERF in humans and LGSGTLGRF in mice and rats have been detected. We found that NMR contains the conserved metalloprotease recognition sequences PPLPENQPLE at its potential β-cut site and LGPETLGRF at its potential α-cut site. The latter is less well conserved. It shares total similarity with human besides the first amino acid in the sequence, which however is similar to the rat and mouse amino acid sequences (Supplementary Fig. 3). This indicates that membrane-bound Klotho in NMR, like rats, mice and humans, can be cleaved by sheddases, potentially resulting in forming full-length soluble Klotho (α-cut) or Kl1 and KL2 soluble Klotho fragments (both α and β-cut).

## Discussion

The discovery by Kuro-o in 1997 of Klotho as an “anti-aging protein” has had a significant impact on our current understanding of the aging process^13,24^. The present investigation was primarily designed to examine whether the remarkable longevity of the NMR might partially be due to elevated *klotho* expressed in the kidney. Thus, the present comparative study examined for the first time the levels of *klotho* mRNA in kidneys of NMR compared to RN and demonstrated that *klotho* was expressed in the kidneys of NMR at a level similar to that of RN.

In 2012 the genome for NMR (Broad Institutr HetGal_Female 1.0/ hetgla2) was sequenced, and predicted gene sequences for *klotho* have been identified in the NMR (XM_021251513.1, NCBI) with three exons and two introns and a 3000 bp coding region. However, in Ensemble the same genome assembly for NMR predicted gene for *klotho* was shown to contain 6 exon and 5 introns (2763 bp) (KL-201 ENSHGLT00000007403.1). *Klotho* is flanked by *PDS5B* and *STARD13* as seen in other species, including humans, rats, and mice^16^. The chromosome location of *Kl* in NMR is unknown. The *sKL* contains a 50 bp insertion between exon 3 and 4 in human *Kl*^17^. *sKl* occurs in human and mice due to alternative splicing and has not been detected in rats^16^.

As in the present investigation the levels of *klotho* expressed in the kidneys of NMR and RN were equal the existence of a potential extrarenal expression *of mKl* was further examined in other organs of NMR, such as the lungs, skin, and liver. It was found that *mKl* was not expressed in the skin or the pulmonary tissue of NMR, while a highly significant expression of *mKl* was demonstrated in the liver of NMR.

A large piece of the amplified and sequenced *mKl* in the liver and its alignment to kidney *mKl*, confirmed the expression of *mKl* in the NMR liver. Furthermore, the presence of the *sKl* in the liver of NMR was excluded. Amplification and sequencing of the predicted region of alternative splicing, which potentially could result in nonsense-mediated decay of mRNA as demonstrated in both humans and mice^17^ was performed, and it was shown that NMR hepatic *klotho* did not include the 50 bp introns, which have been shown in the alternatively spliced region.

The *mKl* codes for a transmembrane Klotho, a type-1 single-pass protein, with a long extracellular region consisting of two separate glucosyl hydrolase domains, repeats Kl1 and Kl2, which are connected to a short 20 amino acid single transmembrane domain. Cleavage of Klotho generates two soluble circulating forms^19,20^. Metalloproteases, mainly ADAM 10 and ADAM 17, have previously been shown to be responsible for Klotho shedding^19,20^. In silico analysis of the sequence of the NMR *klotho* gene as well as amino acids show the existence of similar secretase recognition cleavage sites, as seen in humans, mice and rats. Sanger sequencing of NMR liver and kidney *klotho* confirmed the existence of recognition sites for both α and β-cut cleavage (Supplementary Fig. 2). This indicates that NMR Klotho potentially is shedded into the circulation. Whether this results in increased plasma levels and contributes to extend life span in the NMR needs to be further examined.

In mice circulating Klotho is primarily derived from the kidney, as deletion of kidney *klotho* resulted in a phenotype similar to that of total *klotho* knockout. Unfortunately, no specific commercial Klotho antibodies for NMR are at present available (Supplementary Fig. 4). In order to further characterize the hepatic expression of Klotho in NMR, we used proteomics as the Parallel Reaction Monitoring analysis and detected the Klotho peptides in the liver of NMR similar to that in the kidney. Whether the liver Klotho in NMR contributes to circulating hormonal levels remains to be examined.

The results of the present investigation clearly indicated that Klotho in NMR was expressed both in the kidney and also extrarenally in the liver, which was not the case in RN.

Thus, the present investigation is the first clearly to demonstrate a differential tissue expression of the *klotho* gene in different species. Our results underline the need for a thorough examination of experimental models used for differential local tissue expression of *klotho* in order to ensure, whether a role for Klotho exists, as previously discussed by our and other groups considering a vascular role of Klotho^23^.

Essential differences between NMR and other mammals have been demonstrated by a cross-species analysis^49^. Heinze et al. showed that the liver of NMR harbors several significant features, which contrast that of a short-lived closely related rodent, the guinea pig, including a high reliance on fatty acids for energy yield, gained from an increased abundance of enzymes responsible for lipid turnover^49^. A recent study has shown that Klotho might have a pro-lipolytic effect in obese mice, and that Klotho affects the regulation of energy metabolism^50^. It may be hypothesized that the extrarenal expression of Klotho in the liver of NMR might have a local regulatory effect and that the longevity of NMR might be favored by a high level of lipid oxidation.

Another interesting aspect which might link the expression of Klotho in the liver of NMR to longevity is that NMR is highly adapted to very harsh living conditions. NMRs live in dark underground tunnel systems (up to two meters under the surface) with low oxygen and high carbon dioxide^51^. They are exposed to a high level of both extracellular and intracellular oxidative damage^51,52^. The NMR has developed profound pathways for the removal of these factors of accumulative oxidative stress, and NMR is considered being resilient to oxidative stress, despite a high production of reactive oxygen species (ROS) from cytoplasmic and mitochondrial sources^9,53^. These metabolic pathways likely contribute to NMR’s healthy and successful aging process. ROS-sensitive apoptosis signal-regulating kinase 1 (ASK1)-signalosome is a mediator of cell senescence and the aging phenotype of the *klotho* knockout mice model^54^. Klotho deficiency in vivo is associated with decreased hepatic nuclear and cytoplasmic levels of nuclear factor erythroid 2-related factor 2 (Nrf2), and an inverse correlation has been demonstrated between Klotho and Nrf2^54^. Overexpression of *klotho* results in nuclear translocation of Nrf2 and activation of the antioxidant response element (ARE) in the promoter of antioxidant genes^54,55^. We hypothesize that NMR presumably uses its extrarenal hepatic Klotho to reduce the generation of ROS, which arise from mitochondria and cytosol in the liver. This is likely mediated by enhanced Nrf2 and its antioxidant enzyme network, which have been found to be to be elevated in the liver of NMR^49,52,53,56,57^.

Low P upregulates the expression of Klotho and enhances autophagy independently. The link between P toxicity, Klotho and, autophagy^58,59^ is interesting, and it might be assumed that the extrarenal production of Klotho in the liver of NMR and the low plasma P^60^ might be involved in elevated autophagy and the prolonged life span in NMR^61^. NMR has a low plasma P^60^, which potentially can induce an increased autophagy flux e.g. by disturbing the binding between beclin1 (BCL1) and its negative regulator (BCL2)^58^. Experimental and clinical observations provide strong evidence for a toxic effect of high P, which accelerates the aging process and implies a role for P homeostasis as a toxic factor in the mammalian aging process similar to that of knockout of *klotho*^62^. Thus, long-term intake of high P is associated with decreased renal expression of Klotho, low circulating Klotho, and both increased PTH and FGF23^63^. Therefore, a defect in Klotho causes not only P retention, but also a premature-aging syndrome in mice, which can be rescued by resolving the hyperphosphatemia^64^. As previously mentioned, a comparative longevity analysis has revealed existence of a significant inverse correlation between plasma P and lifespan in different mammals. Interestingly plasma P levels of the NMR is half that of mice and rats^40,65^.

### In summary

The results of the present investigation clearly demonstrate that in the Naked Mole Rat with an extreme longevity, the expression of *klotho* in the kidneys is at the same level, as that found in the common rats, RN. Besides, for the first time, it is shown that the Naked Mole Rats exhibit a highly significant extrarenal expression of Klotho in the liver, a finding which is in contrast to no liver expression of Klotho in Rattus Norvegicus. Thus, differential tissue expression of Klotho in different species is demonstrated. The results of the present comparative study indicate that the longevity-related gene, *klotho*, which in the Naked Mole Rat also is expressed in the liver, might have a specific function as a further longevity and health-span-expanding factor.

## Supplementary Information

Supplementary Figures.
