# Supplementary material for: Extrarenal expression of α-klotho, the kidney related longevity gene, in Heterocephalus glaber, the long living Naked Mole Rat
Source: Sci Rep. 2021 Jul 28;11:15375. doi: 10.1038/s41598-021-94972-1 (PMC8319335; doi:10.1038/s41598-021-94972-1)

# Supplementary Figures

## Extrarenal expression of *α-klotho,* the kidney related longevity gene, in *Heterocephalus glaber,* the long living Naked Mole Rat.

by

Morevati M, Mace ML, Egstrand S, Nordholm A, Doganli C, Strand J,
Rukov JL, Torsetnes SB, Gorbunova V, Olgaard K, Lewin E

**Correspondence**:
Marya Morevati, PhD,
Nephrological Department P 2131,
Rigshospitalet,
9 Blegdamsvej,
2100 Copenhagen,
Denmark.
Phone: +45 35452777
Mobile Phone: +45 50467285
E-mail: marya.morevati@regionh.dk

**Supplementary Figure 1**

**Expression of a long fragment of the *Klotho* mRNA in Naked Mole Rat liver and kidney.**

Expression of a long fragment of the *Kl* mRNA (2357) in NMR liver and kidney is displayed on an agarose gel, which is further used for sequencing. Each line represents a pool of samples from five Naked Mole Rats; (n=5)

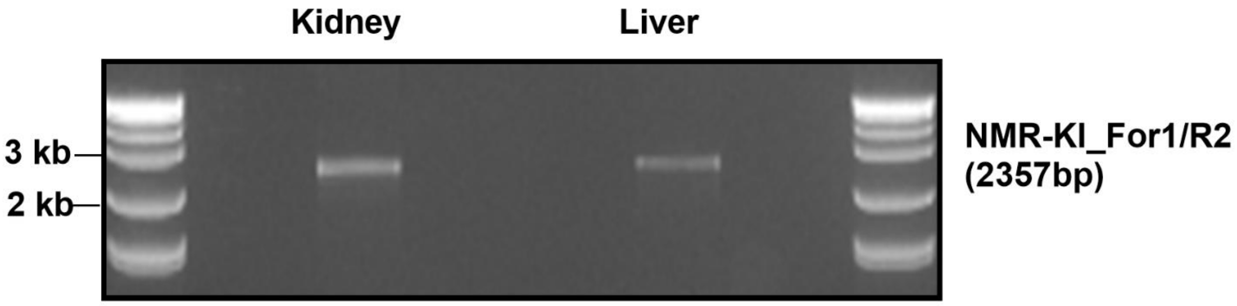


**Supplementary Figure 2**

**Alignment of partially sequenced Klotho in the kidney and liver of the Naked Mole Rat.**

The long-amplified fragment of mKl mRNA (2357) was partially sequenced in the kidney and liver of Naked Mole Rats by Sanger Sequencing. Detected pieces for mKl in the kidney were aligned to the pieces detected from the liver, and non sequenced parts were filled out by reference sequences for Klotho obtained from (XM_021251513.1).

Each sample is a pool of five livers or kidneys from Naked Mole Rats.

Text in black (Bold): sequenced kidney mKl;

Text in red (Bold): sequenced liver mKl;

|: matched sequence;

-: gap between sequences;

Empty space between the sequences: mismatches.

NMR: Naked Mole Rat;

mKl: membrane-bound α-klotho.

The two fragments, which code for the potential cleavage sites of Klotho in the Naked Mole Rat kidney and liver, “PPLPENQPL” and “LGPETLGRF”, are presented in yellow.

```
ATGGCTAGCAGTGATAAAGCAGGATTATCTCCGCTGCCCTCTGAGGGATCTCTTACTCCT  60
||||||||||||||||||||||||||||||||||||||||||||||||||||||||||||
ATGGCTAGCAGTGATAAAGCAGGATTATCTCCGCTGCCCTCTGAGGGATCTCTTACTCCT  60

GATGTCCCGAATAAAGGGGTCCGCGCAGCATGCCCGCCCGCGTCGCCCGCGGCCGCCGCT  120
||||||||||||||||||||||||||||||||||||||||||||||||||||||||||||
GATGTCCCGAATAAAGGGGTCCGCGCAGCATGCCCGCCCGCGTCGCCCGCGGCCGCCGCT  120

GCCACCACTGCTCCTGCTGCTGGCCCTGAGCCCGGAGACGGCGCGCAGACCTGGNGCCGG  180
||||||||||||||||||||||||||||||||||||||||||||||||||||||||||||
GCCACCACTGCTCCTGCTGCTGGCCCTGAGCCCGGAGACGGCGCGCAGACCTGGNGCCGG  180

CTTCGGCGTCCTGCGGTCCCTGACGCCGCTGGCCTCCACGATACTTTCCCCGACCGCTTC  240
||||||||||||||||||||||||||||||||||||||||||||||||||||||||||||
CTTCGGCGTCCTGCGGTCCCTGACGCCGCTGGCCTCCACGATACTTTCCCCGACCGCTTC  240

CTCTGGGCCGTGGGCAGCGCCGCCTACCAGCCCGAAGGTGGCTGGAGGCAGCACGCCAAG  300
||||||||||||||||||||||||||||||||||||||||||||||||||||||||||||
CTCTGGGCCGTGGGCAGCGCCGCCTACCAGCCCGAAGGTGGCTGGAGGCAGCACGCCAAG  300

GGCGCGTTCATCTGGGACACGTTCACCCATCGGCCCCCGGGTGCCCAGTCACCTTTCCCC  360
||||||||||||||||||||||||||||||||||||||||||||||||||||||||||||
GGCGCGTTCATCTGGGACACGTTCACCCATCGGCCCCCGGGTGCCCAGTCACCTTTCCCC  360

CCTGCCACTGGGGATGTGGCCAGCGACGGCTACAACAATTTCTTCCGAGACACCGAGAGG  420
||||||||||||||||||||||||||||||||||||||||||||||||||||||||||||
CCTGCCACTGGGGATGTGGCCAGCGACGGCTACAACAATTTCTTCCGAGACACCGAGAGG  420

CTACGCGAGCTTGGGGTCATCCACTGCCGCTTCTCCATCTCGTGGGCGCGGGTGTTTCCC  480
||||||||||||||||||||||||||||||||||||||||||||||||||||||||||||
CTACGCGAGCTTGGGGTCATCCACTGCCGCTTCTCCATCTCGTGGGCGCGGGTGTTTCCC  480

AATGGCAGCGCGGGCGCCCCCAACCTCGAGGGCCGGCGCTATGCTACTACCGGCACCTGA  540
||||||||||||||||||||||||||||||||||||||||||||||||||||||||||||
AATGGCAGCGCGGGCGCCCCCAACCTCGAGGGCCGGCGCTATGCTACTACCGGCACCTGA  540

GGGAGAGGCTGCAGGAGCTGGGCCAACCGTGACCTGGCCGACCACTTCAGGGATTATGCC  600
||||||||||||||||||||||||||||||||||||||||||||||||||||||||||||
GGGAGAGGCTGCAGGAGCTGGGCCAACCGTGACCTGGCCGACCACTTCAGGGATTATGCC  600

GAGCTCTGCTTCCGCCACTTCGGCGGCCAAGTCAAATACTGAATCACGGTCAACAACTCC  660
||||||||||||||||||||||||||||||||||||||||||||||||||||||||||||
GAGCTCTGCTTCCGCCACTTCGGCGGCCAAGTCAAATACTGAATCACGGTCAACAACTCC  660

TACGTAGTGGTCTGGCACTGCTATGCTGCTGGGCGCCTGGCCCCGGGAGTCTGGGGCAGC  720
```

```
||||||||||||||||||||||||||||||||||||||||||||||||||||||||||||
TACGTAGTGGTCTGGCACTGCTATGCTGCTGGGCGCCTGGCCCCGGGAGTCTGGGGCAGC   720

CCGCGGCTCGGGTACCTGGTGGCGCACAACCTTCTTCTGGCTCATGCCAAAATCTGGCAT   780
||||||||||||||||||||||||||||||||||||||||||||||||||||||||||||
CCGCGGCTCGGGTACCTGGTGGCGCACAACCTTCTTCTGGCTCATGCCAAAATCTGGCAT   780

CTCTACAATACTTCCTTCCGTCCAACTCAGGGAGGCCAGGTGTCCATTGCCCTGGGTTCC   840
||||||||||||||||||||||||||||||||||||||||||||||||||||||||||||
CTCTACAATACTTCCTTCCGTCCAACTCAGGGAGGCCAGGTGTCCATTGCCCTGGGTTCC   840

CACTGGATCACTCCTCGAAGAATGACTGACCACAACATCAAAGAATGTCAAAAATCTCTT   900
||||||||||||||||||||||||||||||||||||||||||||||||||||||||||||
CACTGGATCACTCCTCGAAGAATGACTGACCACAACATCAAAGAATGTCAAAAATCTCTT   900

GACTTTGTCCTTGGCTGGTTTGCCAAACCCATATTTATTGGTGATGATTATCCTGATGAC   960
||||||||||||||||||||||||||||||||||||||||||||||||||||||||||||
GACTTTGTCCTTGGCTGGTTTGCCAAACCCATATTTATTGGTGATGATTATCCTGATGAC   960

ATGAAGAGTAACCTTTCATCTCTTCTGCCTGATTTTACTGAATCTGAGAAAAAGTTCATC   1020
||||||||||||||||||||||||||||||||||||||||||||||||||||||||||||
ATGAAGAGTAACCTTTCATCTCTTCTGCCTGATTTTACTGAATCTGAGAAAAAGTTCATC   1020

AAGGGAACAGCTGACTTTTTTTCACTTTCCTTTGGACCAACATTGAGCTTTCAGCTATTG   1080
||||||||||||||||||||||||||||||||||||||||||||||||||||||||||||
AAGGGAACAGCTGACTTTTTTTCACTTTCCTTTGGACCAACATTGAGCTTTCAGCTATTG   1080

GAGCCCCACATGAAGTTCCACCAACTAGAATTTCCCAGCCTGAGGCAACTCCTCTCCTGG   1140
||||||||||||||||||||||||||||||||||||||||||||||||||||||||||||
GAGCCCCACATGAAGTTCCACCAACTAGAATTTCCCAGCCTGAGGCAACTCCTCTCCTGG   1140

ATTGACCTTGAATATAATCATCCTGAAATATTTATTGTGGAAAGTGGCTGGTTTGTCTCA   1200
||||||||||||||||||||||||||||||||||||||||||||||||||||||||||||
ATTGACCTTGAATATAATCATCCTGAAATATTTATTGTGGAAAGTGGCTGGTTTGTCTCA   1200

GGGACCACCAAGAGAGATGATGCCAAATATATGTATTTACCTAAAAAAGTTCGTAATGGA   1260
||||||||||||| |||||   || |||||||||| ||||||||||||||||||||||||
GGGACCACCAAGAAAGATGGCACCGAATATATGTA-TTACCTAAAAAAGTTCGTAATGGA   1259

AAGCTTAAAAGCCATCAGGCTGGATGGGGTTGATGTCATTGGGTACACAGCATGGTCCCT   1320
||||||||||||||||||||||||||||||||||||||||||||||||||||||||||||
AAGCTTAAAAGCCATCAGGCTGGATGGGGTTGATGTCATTGGGTACACAGCATGGTCCCT   1319

CATGGATGGTTTCGAATGGCACAGAGGCTACAGCATCCGACGTGGACTCTTCTATGTCGA   1380
||||||||||||||||||||||||||||||||||||||||||||||||||||||||||||
CATGGATGGTTTCGAATGGCACAGAGGCTACAGCATCCGACGTGGACTCTTCTATGTCGA   1379

CTTTTTGAGCCAGGATAAGAAGTTGTTGCCAAAGTCTTCAGCCTTGTTCTACCAAAAGCT   1440
||||||||||||||||||||||||||||||||||||||||||||||||||||||||||||
CTTTTTGAGCCAGGATAAGAAGTTGTTGCCAAAGTCTTCAGCCTTGTTCTACCAAAAGCT   1439
```

```
GATAGAGAGCAATGGCTTCCCTCCTTTACCTGAAAACCAACCCCTAGAAGGGACATTTCC   1500
||||||||||||||||||||||||||||||||||||||||||||||||||||||||||||
GATAGAGAGCAATGGCTTCCCTCCTTTACCTGAAAACCAACCCCTAGAAGGGACATTTCC   1499

CTGTGACTTTGCTTGGGGAGTTGTTGACAACCACATTCAAGTAGACACCACTCTGTCTCA   1560
||||||||||||||||||||||||||||||||||||||||||||||||||||||||||||
CTGTGACTTTGCTTGGGGAGTTGTTGACAACCACATTCAAGTAGACACCACTCTGTCTCA   1559

GTTTACCGACACCAACGTTTACCTGTGGGACGTCCATCACAGTAAGAGGCTTATTAAAGT   1620
|||||||||||||||||||||||||||||||||||||||||||||||||||||||||||
GTTTACCGACACCAACGTTTACCTGTGGGACGTCCATCACAGTAAGAGGCTTATTAAAGT   1619

GGATGGAGTTGCAGCCAAGAAGCAGAAATCCTACTGTGTTGCTTTCACTGCCATCTGGCC   1680
||||||||||||||||||||||||||||||||||||||||||||||||||||||||||||
GGATGGAGTTGCAGCCAAGAAGCAGAAATCCTACTGTGTTGCTTTCACTGCCATCTGGCC   1679

CCAGATAGCCCTACTGCAGGAAATGCACGTCACGCATTTTCACTTCTCTCCGGATTGGGC   1740
|||||| ||||||||||||||||||||| ||| ||||||||||||||||| || || |||
CCAGATTGCCCTACTGCAGGAAATGCACATCAGGCATTTTCACTTCTCTCTGGGTTAGGC   1739

GTTAATCCTCCCCCTGGGTAACCTGTCCCAGGTGAACAGCACAGTCCTGCGCTACTACGG   1800
|||||||||||||||||||||||||||||||||||||||||||||| ||| ||||||| |
TTTAATCCTCCCCCTGGGTAACCTGTCCCAGGTGAACAGCACAGTCTTGCACTACTACCG   1799

CTGCGTGGTCAGTGAGCTGCTCTGCGCCAACATCACGCCTGTGGTAGCCCTGTGGCAGCC   1860
||| |||||||||||||||||||| | |||||||| ||||| ||||||||||||||||||
CTGTGTGGTCAGTGAGCTGCTCTGTGTCAACATCAAGCCTGGGGTAGCCCTGTGGCAGCC   1859

TGCGGCCCAGCACCACGGCCTGCCAGGTCCCCTGGCAAAGCAGGGAGCCTGGGAGAACCC   1920
|||||||||||||||| ||||||||||||||||||| ||||||||||||||||||||| |
TGCGGCCCAGCACCACAGCCTGCCAGGTCCCCTGGCCAAGCAGGGAGCCTGGGAGAACAC   1919

TCGCACTGCCCTGGCCTTTGCAGAGTACGCCAGACTCTGCTTTGAAGAGCTGGGCCACTA   1980
| |||| ||||||||||||| ||||||| ||||||||||||||||||||||||||||| |
TTGCACCGCCCTGGCCTTTGTAGAGTACACCAGACTCTGCTTTGAAGAGCTGGGCCACCA   1979

CGTCGAGTTCTGGATCACGATGAACGAGCCGTACATGCGCAACCTGACCTACCGCGCGGG   2040
| || |||||||||||||||||||||||||||||||||||||||||||||||||||||||
CATCAAGTTCTGGATCACGATGAACGAGCCGTACATGCGCAACCTGACCTACCGCGCGGG   2039

GCATCACCTTCTGAAGGCTCACGCACTGGCTTGGCGCCTGTATGATGAAAAGTTTAGACC   2100
||||||||||||||||||||||||||||||||||||||||||||||||||||||||||||
GCATCACCTTCTGAAGGCTCACGCACTGGCTTGGCGCCTGTATGATGAAAAGTTTAGACC   2099

CACCCAGAAAGGTAAAATATCCATAGCTTTACAGGGTGATTGGATAGAACCAGCCTGCCC   2160
||||||||||||||||||||||||||||||||||||||||||||||||||||||||||||
CACCCAGAAAGGTAAAATATCCATAGCTTTACAGGGTGATTGGATAGAACCAGCCTGCCC   2159

TTTGTCCCAAAAGGACAAGGAAGTGGCCGAGAGAGTGTTGGAATTTGACATTGGCTGGCT   2220
```

```
||||||||||||||||||||||||||||||||||||||||||||||||||||||||||||
TTTGTCCCAAAAGGACAAGGAAGTGGCCGAGAGAGTGTTGGAATTTGACATTGGCTGGCT  2219

GGCTGAAAAGCCCATATTCGGCTCTGGGGATTACCCACGCGTGATGAGGGACTGGCTGAA  2280
|||||||||||||||||||||||||||||||||||||||||||||||||||| ||||| ||
GGCTGAAAAGCCCATATTCGGCTCTGGGGATTACCCACGCGTGATGAGGGA-TGGCT-AA  2277

CCAAAGAAGCAATTTTCTTCTCCCTTTTTTCACTGAAGAGGaaaaaaaGTTAATCCAAGT  2340
||||||||||||||||||||||||||||||||||||||||||||||||||||||||||||
CCAAAGAAGCAATTTTCTTCTCCCTTTTTTCACTGAAGAGGAAAAAAAGTTAATCCAAGT  2337

TTCCTTTGACTTTTTAGCCCTAAGCCATTACACCACCATCCTTGTCGACTGGGAAAAAGA  2400
||||||||||||||||||||||||||||||||||||||||||||||||||||||||||||
TTCCTTTGACTTTTTAGCCCTAAGCCATTACACCACCATCCTTGTCGACTGGGAAAAAGA  2397

AGATCCCTTAAAATACAACGACTACCTAGAAGTGCAAGAAATGACTGACATCACTTGGCT  2460
||||||||||||||||||||||||||||||||||||||||||||||||||||||||||||
AGATCCCTTAAAATACAACGACTACCTAGAAGTGCAAGAAATGACTGACATCACTTGGCT  2457

CAACTCCCCCAGCCAGGTGGCAGTGGTGCCCTGGGGGCTGCGCAGAGTGCTGAACTGGCT  2520
||||||||||||||||||||||||||||||||||||||||||||||||||||||||||||
CAACTCCCCCAGCCAGGTGGCAGTGGTGCCCTGGGGGCTGCGCAGAGTGCTGAACTGGCT  2517

GAAGATAAAGTACGGAGACCTCCCTGTATATAATTGCCAATGGGATCGACGACGATCCAC  2580
||||||||||||||||||||||||||||||||||||||||||||||||||||||||||||
GAAGATAAAGTACGGAGACCTCCCTGTATATAATTGCCAATGGGATCGACGACGATCCAC  2577

ACGAGGAGCAAGACAAGCTGAGGATGTATTACCTACAAAATTATGTAAACAAAGCTCTGA  2640
||||||||||||||||||||||||||||||||||||||||||||||||||||||||||||
ACGAGGAGCAAGACAAGCTGAGGATGTATTACCTACAAAATTATGTAAACAAAGCTCTGA  2636

AAGCTTATGTATTGGACGGTATCAATCTTTGTGGATACTTTGCTTATTCATTTAATGATC  2700
||||||||||||||||||||||||||||||||||||||||||||||||||||||||||||
AAGCTTATGTATTGGACGGTATCAATCTTTGTGGATACTTTGCTTATTCATTTAATGATC  2695

GCTCAGCTCCGAAGTTTGGCTTCTATCGTTATGCTGCAAATCAGTTTGAGCCGAAACCAT  2760
||||||||||||||||||||||||||||||||||||||||||||||||||||||||||||
GCTCAGCTCCGAAGTTTGGCTTCTATCGTTATGCTGCAAATCAGTTTGAGCCGAAACCAT  2755

CCATGAAACATTACAGGAAAATTATTGACAACAACGGTTTCCTGGGCCCTGAAACTCTGG  2820
||||||||||||||||||||||||||||||||||||||||||||||||||||||||||||
CCATGAAACATTACAGGAAAATTATTGACAACAACGGTTTCCTGGGCCCTGAAACTCTGG  2815

GAAGGTTTTGCCCAGAAGATTATACCATGTGCACTGAATGCAGCTTCTTTCACACCCGAA  2880
|||||||||||||||||||||||||||||| ||||||||||||||||||||||||| |||
GAAGGTTTTGCCCAGAAGATTATACCATGTACACTGAATGCAGCTTCTTTCACACCTGAA  2875
```

**Supplementary Figure 3**

**Alignment of Naked Mole Rat Klotho amino acid sequence**

**(Query_5384: ENSHGLP00000007318 Ensembl Translation) with**

**Rattus Norvegicus (BAA34740.1: GenBank).**

The expended view of the Klotho amino acid sequence showing residue conservation, red for identical residues, blue for no gaps, gray is containing gaps.

The two cleavage sites “PPLPENQPL” and “LGPETLGRF”, which potentially are leading to the shed, circulating forms of Naked Mole Rat Klotho protein are underlined in yellow.

The two peptides found by proteomics analysis in the present investigation (Figure 5) are highlighted in the green boxes in the protein sequence.

```
Query_5384   1    MPARAPPR-FPRPPLPPLL----LLLALGGRCLRAEPGDGAQTWARFARPPVPESAGLLHDTFPDGFLWAVGSAAYQTEG  75
BAA34740.1   1    MPARAPPRRLPRLLLLRLLSLHLLLLTLRARCLSAEPGQGAQTWARFARPPVPEASGLLHDTFPDGFLWAVGSAAYQTEG  80

Query_5384   76   GWRQHGKGASIWDTFTHRSPA-------------APSPFPAATGDVASDGYNNVFRDTEGLRELGVTHYRFSISWARVFP  142
BAA34740.1   81   GWRQHGKGASIWDTFTHHPRAIPEDSPIVMAPSGAPLPPLPSTGDVASDSYNNVYRDTEGLRELGVTHYRFSISWARVLP  160

Query_5384   143  NGSAGAPNREGLRYYRRLLERLRELGVQPVVTLYHWDLPQSLQDVYGGWANRALADHFRDYAELCFRHFGGQVKYWITID  222
BAA34740.1   161  NGTAGTPNREGLRYYRRLLERLRELGVQPVVTLYHWDLPQRLQDTYGGWANRALADHFRDYAELCFRHFGGQVKYWITID  240

Query_5384   223  NPYVVAWHGYATGRLAPGVRGSPRLGYLVAHNLLLVSAR-----------------------------------------  261
BAA34740.1   241  NPYVVAWHGYATGRLAPGVRGSSRLGYLVAHNLLLAHAKVWRLYNTSFRPTQGGRVSIALGSHWITPRRMTDYHIRECQK  320

Query_5384   262  -----------------DQPKG------------HPGEREDLRGWFFDFHIFVSP--DFLLLLTS----NFISLCLRQLL  306
BAA34740.1   321  SLDFVLGWFAKPIFIDGDYPKSMKNNLSSLLPDFTESEKRFIRGTADFFALSFGPTLSFQLLDPSMKFRQLESPSLRQLL  400

Query_5384   307  SWIDLEYNHPEIFIVESGWFVSGTTKRDDAKYMYYLKKFVMESLKAIRLDGVDVIGYTAWSLMDGFEWHRGYSIRRGLFY  386
BAA34740.1   401  SWIDLEYNHPQIFIVENGWFVSGTTRRDDAKYMYYLKKFIMESLKAIRLDGVDVIGYTAWSLMDGFEWHRGYSIRRGLFY  480

Query_5384   387  VDFLSQDKKLLPKSSALFYQKLIESNGFPPLPENQPLEGTFPCDFAWGVVDNHIQVDTTLSQFTDTNVYLWDVHHSKRLI  466
BAA34740.1   481  VDFLSQDKELLPKSSALFYQKLIENNGFPPLPENQPLEGTFPCDFAWGVVDNYIQVDPTLSQFTDPNVYLWDVHHSKRLI  560

Query_5384   467  KVDGVAAKKRKSYCVDFAAIRPQIALLQEMHVTHFHFSLDWALILPLGNLSQVNSTVLRYYGCVVSELLCANITPVVALW  546
BAA34740.1   561  KVDGVVAKKRKPYCVDFSAIRPQITLLREMRVTHFRFSLDWALILPLGNQTQVNRTVLHFYRCMVSELVHANITPVVALW  640

Query_5384   547  QPAAQHHGLPGPLAKQGAWENPRTALAFAEYARLCFEELGHYVEFWITMNEPYMRNLTYRAGHHLLKAHALAWRLYDEKF  626
BAA34740.1   641  QPATPHQGLPHALAKHGAWENPHTALAFADYANLCFEELGHWVKFWITINEPNSRNMTYRAGHHLLKAHALAWHLYDDKF  720

Query_5384   627  RPTQKGKISIALQADWIEPACPLSQKDKEVAERVLEFDIGWLAEPIFGSGDYPRVMRDWLNQRSNFLLPFFTEEEKKLIQ  706
BAA34740.1   721  RAAQKGKISIALQVDWIEPACPFSQKDKEVAERVLEFDVGWLAEPIFGSGDYPHVMREWLNQKNNFLLPYFTEDEKKLIR  800

Query_5384   707  GSFDFLALSHYTTILVDWEKEDPLKYNDYLEVQEMTDITWLNSPSQVAVVPWGLRRVLNWLKIKYGDLPVYIIANGIDDD  786
BAA34740.1   801  GSFDFLALSHYTTILVDWEKEDPIKYNDYLEVQEMTDITWLNSPNQVAVVPWGLRKALNWLRFKYGDLPMFVTANGIDDD  880

Query_5384   787  PHEEQDKLRMYYLQNYVNEALKAYVLDGINLCGYFAYSFNDRSAPKFGFYRYAANQFEPKPSMKHYRKIIDNNGFLGPET  866
BAA34740.1   881  PHAEQDSLRMYYIKNYVNEALKAYVLDGINLCGYFAYSLSDRSVPKSGFYRYAANQFEPKPSIKHYRKIIDNNGFLGSGT  960

Query_5384   867  LGRFCPEDYTMCTECSFFHTRKSLLAFIVFLFFAFIVSLSLMFYYSKKGRRSYK  920
BAA34740.1   961  LGRFCPEEYTVCTGCGFFQTRKSLLAFISFLVFAFVTSLALIYYYSKKGRRRYK  1014
```

**Supplementary Figure 4**

**Antibody for detection of Klotho in Rattus Norvegicus is not suitable for detection of Klotho in the Naked Mole Rat.**

The KO604 (Colne no KM2119) antibody was used to detect Klotho (KL) in the kidney of Rattus Norvegicus and examined whether the antibody was able to detect Klotho in the Naked Mole Rat. The antibody detected Klotho as expected at 130 KDa in the kidney of Rattus Norvegicus, but failed to detect the Klotho protein in the kidney of the Naked Mole Rat, where Klotho otherwise was shown at the level of mRNA as well as the protein level by proteomics analysis.

One explanation might be that this could be due to a potential different folding of the Klotho protein in Naked Mole Rat, which thereby makes the recognizing epitope not available for the (Clone no KM2119) antibody. Another potential explanation could be due to post translational modification of Klotho, such as methylation or glycosylation at the binding site of antibody.

Klotho was measured by Western blot analysis, and PARK7 was used as housekeeping protein. (n=5).

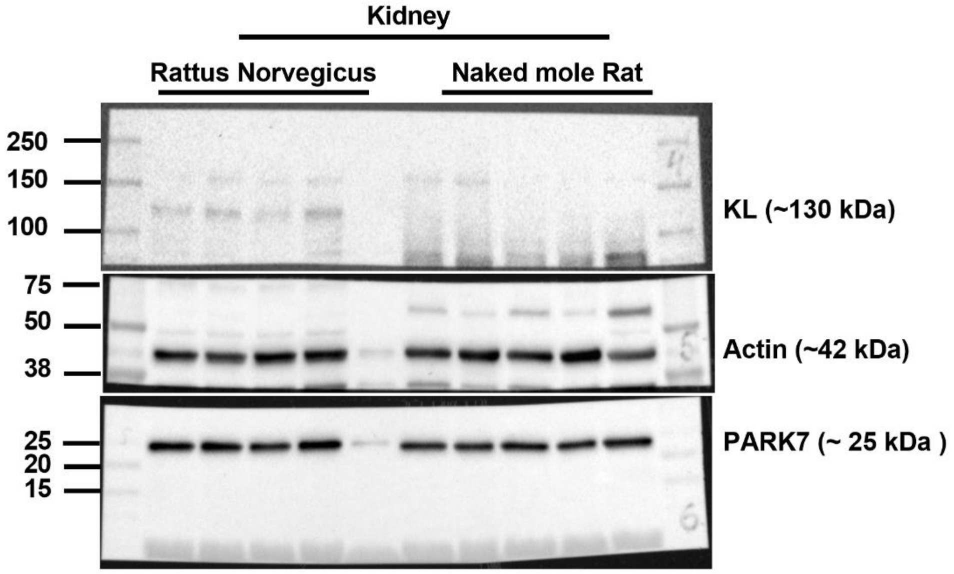

**Supplementary Figure 5**

**Uncropped agarose gels.**

(a) from Figure 1

(b1) and (b2) from Figure 3a,

(c) from Figure 4

(d) from Supplementary Figure 1.

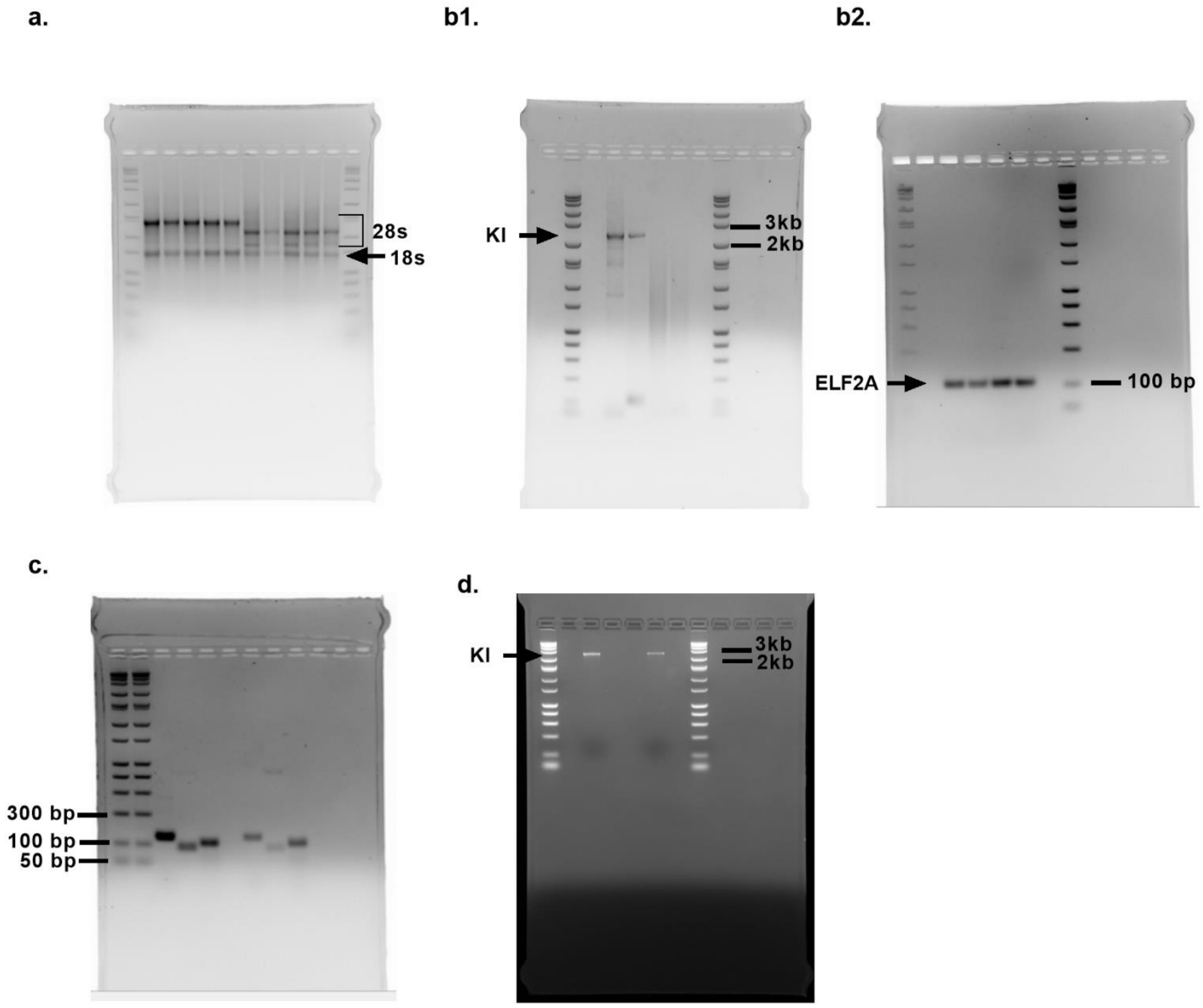

Supplement: Supplementary file 1 — Supplementary Figures. [file 41598_2021_94972_MOESM1_ESM.pdf]
